# Quorum sensing in biofilm-mediated heavy metal resistance and transformation: environmental perspectives and bioremediation

**DOI:** 10.3389/fmicb.2025.1607370

**Published:** 2025-07-03

**Authors:** Subhoshmita Mondal, Alice Melzi, Sarah Zecchin, Lucia Cavalca

**Affiliations:** Dipartimento di Scienze per gli Alimenti, la Nutrizione e l’Ambiente (DeFENS), Università degli Studi di Milano, Milan, Italy

**Keywords:** heavy metal, quorum sensing, biofilm, extracellular polymeric substances, bioremediation

## Abstract

Quorum sensing is a fundamental mechanism of bacterial cell-to-cell communication that enables microbial communities to adapt to environmental stresses. Although the role of quorum sensing in biofilm formation and heavy metal resistance has been studied across various bacterial species, significant research gaps remain regarding the specific quorum sensing-regulated metabolic pathways involved in heavy metal resistance and transformation, as well as their functional roles in bioremediation. This review provides a comprehensive overview of the connection between quorum sensing and heavy metal resistance and transformation, considering both cellular and ecological perspectives. It highlights recent advancements in understanding quorum sensing-regulated biofilm dynamics and identifies a lack of knowledge related to quorum sensing-mediated heavy metal resistance in natural ecosystems. Furthermore, innovative quorum sensing-based strategies for optimizing bioremediation are explored. By emphasizing the ecological and practical implications of quorum sensing-driven bioremediation, this review aims to contribute to the development of more effective and sustainable approaches for mitigating heavy metal pollution.

## 1 Introduction

Advancements in technology, industrialization and agriculture have significantly increased environmental pollution through the release of toxic wastes and heavy metals (HM), which cause serious ecological and health risks. Globally, the most extensive disposal source of HM is wastewaters. Industrial activities significantly increase the release and accumulation of hazardous HM in the natural environment, threatening the health and yield of natural biomes (Singh et al., 2011; Priyadarshanee and Das, 2021). Over time, HM accumulation in soil significantly impacts agricultural land by reducing fertility and crop productivity. This contamination leads to long-term effects of phytotoxicity at high concentrations, disruption of microbial processes, and the transfer of toxic elements into the human food chain, either through increased crop uptake or ingestion by grazing livestock. After decades of anthropogenic emissions, it has become increasingly difficult to differentiate between naturally occurring and pollution-derived HM concentrations. To address this challenge, various geochemical, statistical, and spatial models, such as enrichment factors, pollution indices, and multivariate analyses, have been developed to quantify anthropogenic contributions to HM accumulation (Bianchini et al., 2012; Yu et al., 2022). These tools are essential for establishing reliable environmental baselines, identifying pollution sources, and informing effective remediation strategies in contaminated ecosystems, particularly in coastal zones where HM pollution is a major concern. In fact, as convergence zones for industrial, agricultural, and urban runoff, coastal areas often exhibit elevated HM concentrations in sediments and aquatic organisms, posing long-term risks to both ecological integrity and human health (El-Sharkawy et al., 2025). This issue is especially critical in Asian and Oceanic countries, where intensive maritime and industrial activities are major pollution sources. With human activity affecting approximately 40% of the world’s coastal waters, global concern over rising anthropogenic HM contamination continues to grow (El-Sharkawy et al., 2025; Bandara and Manage, 2022; Bhuiyan et al., 2025). Therefore, continuous monitoring and robust assessment methodologies remain vital for mitigating environmental and public health risks in these vulnerable ecosystems.

HM contamination levels vary significantly across different countries and regions, influenced by geography, climate, socioeconomic factors, and industrial and agricultural production. Table 1 shows global HM limits in water and soil as per WHO guidelines, US-EPA, and EU directives. In Asiatic countries like China, India and Southeast Asia, HM contamination, particularly from the most threatening metals, has reached alarming levels, exceeding those observed in the United States and Europe. In these regions, atmospheric deposition is a key source of HM input to agricultural soil (Shi et al., 2024).

**Table 1:** Established legal limit concentrations of heavy metals.

HM	Drinking water^*^ (a, b, c)	Surface water^*^ (b)	Public sewage^*^ (b)	Agriculture		Coastal water^*^ (b)	Ground water^*^ (c)
				Irrigation water^*^ (c)	Soil^**^ (c)		
Arsenic	0.05	0.2	0.2	0.2	2.84	0.2	0.01
Mercury	0.001	0.01	0.01	0.01	0.3	0.01	0.006
Cadmium	0.01	2	1	0.01	0.07	2	0.003
Chromium	0.05	0.1	2	0.05	0.32	1	0.05
Selenium	0.01	0.05	0.05	0.04	2	0.05	0.05
Lead	0.1	0.1	1	5.0	1.16	1	0.01
Nickel	0.1	3	3	0.2	0.27	5	0.1
Zinc	15	5	–	15	2.12	15	3
Copper	2	2	–	0.2	0.26	0.0002	1.3

*mg/L, ** mg/kg, a: WHO guidelines (World Health Organization, 2022), b: US-EPA directive (United States Environmental Protection Agency, 2019) c: EU directive (European Union Parliament Directive, 2020).

Globally, agricultural land in China faces significantly high HM pressure. Specifically, annual zinc (Zn) inputs reach 187,742 g/ha—almost nine times higher than Germany (21,237 g/ha per year), while copper (Cu) inputs are over 14 times higher at 71,824 g/ha compared to France (4,869 g/ha), which records the highest annual inputs of Zn and Cu among all the European countries (Hejna et al., 2018). A meta-analysis revealed concerning average peak levels of mercury (Hg), cadmium (Cd), and chromium (Cr) in China’s river systems when compared to Table 1 limits, a problem compounded by the fact that approximately 82% of China’s polluted agricultural soils contain toxic inorganic pollutants such as lead (Pb), Cd, Cr, and As (Wang and Chadwick, 2024; Adnan et al., 2024).

In India, HM pollution significantly exceeds both national and international safety limits (Palit and Hussain, 2022). Between 2019 and 2022, water quality monitoring revealed concerning concentrations of Cd, Cr, and nickel (Ni) (Kumar et al., 2022). Additionally, a study of surficial sediments across Western, Ganga Plains, and Central India reported alarmingly high levels of Cu, Ni, Pb, and Zn (Prasanna et al., 2024).

In European countries HM mostly derive from animal manure, mineral fertilizers and pesticides (Zareh et al., 2024). A recent study estimates the number of potentially contaminated sites in Europe to sum up to 2.5 million, demonstrating the extent of this issue (Dai et al., 2016; Tóth et al., 2016). In 2021, Italy, Germany, and Poland were the top contributors to HM emissions in the EU. Among the most affected areas, the Lombardy region of Northern Italy stands out as the most both industrialized and densely populated, with a Gross Domestic Product (GDP) higher than many EU Member States. According to the Regional Environment Protection Agency (ARPA), between 2017 and 2020, this region experienced consistent HM pollution, with arsenic (As), Cr(VI), iron (Fe), manganese (Mn), Ni, and Zn in groundwater, and As, Cr, and Ni in surface waters (Zanchi et al., 2022).

In the United States, over half of the dairy farms in Wisconsin used feed rations with Cu levels exceeding the recommended guidelines (Hejna et al., 2018; Dai et al., 2016). High Pb levels were detected near urban areas, and peak concentrations of all studied metals occur in the Lower Mississippi River Valley. Spatial prediction data also show high concentrations of Cu, Ni, and Zn in the West Coast, while Cd levels were higher in the central United States (Adhikari et al., 2024).

HM contamination, despite receiving less attention compared to other types of pollution, poses serious threat due to its hazardous effects, even at low concentrations. They enter the human body through ingestion, skin contact, or inhalation, leading to gastrointestinal, respiratory, cardiovascular, reproductive, renal, hemopoietic, carcinogenic, and neurological disorders (Witkowska et al., 2021). HM bind to free thiols or other functional groups, catalyze the oxidation of amino acid side chains, disrupt protein folding, and displace essential metal ions in enzymes, damage cell membranes and DNA.

Certain HM, like Cu, Cr, and Mn, are biologically essential since they are involved in electron transport, enzyme regulation, glucose utilization and protein metabolism, yet become toxic when excessive. In contrast, As, Cd, Pb, and Hg are toxic elements with no biological benefit, causing considerable harm to public health, even when exposure is minimal.

Microbial communities are frequently exposed to HM stress in various environments. Over time, microorganisms have evolved diverse strategies to mitigate HM toxicity and ensure survival. Among these, biofilm formation regulated by quorum sensing (QS) stands out as a protective shield. This review aims to explore the mechanisms underlying biofilm-based HM resistance and transformation by microbial populations at both cellular and ecosystem levels, highlighting the role of QS in HM bioremediation strategies.

## 2 Metal-microbe interaction

Bacteria have been exposed to HM stress for approximately 3.5 billion years. Hence, the resistance mechanisms and genes related to bacterial HM resistance have evolved next to Earth’s development (He et al., 2023). HM toxicity influences microbial communities by changing their structure, size, and diversity. Specifically, variations observed in morphology, cellular metabolism, growth, and nucleic acid composition can lead to cellular membrane disruption (Priyadarshanee and Das, 2021).

Unlike organic pollutants, HM cannot be degraded through chemical or biological processes. Therefore, their transformation into less toxic forms (i.e., bivalent mercury to the less toxic volatile form) or their removal are the only viable remediation strategies.

**Figure 1:**
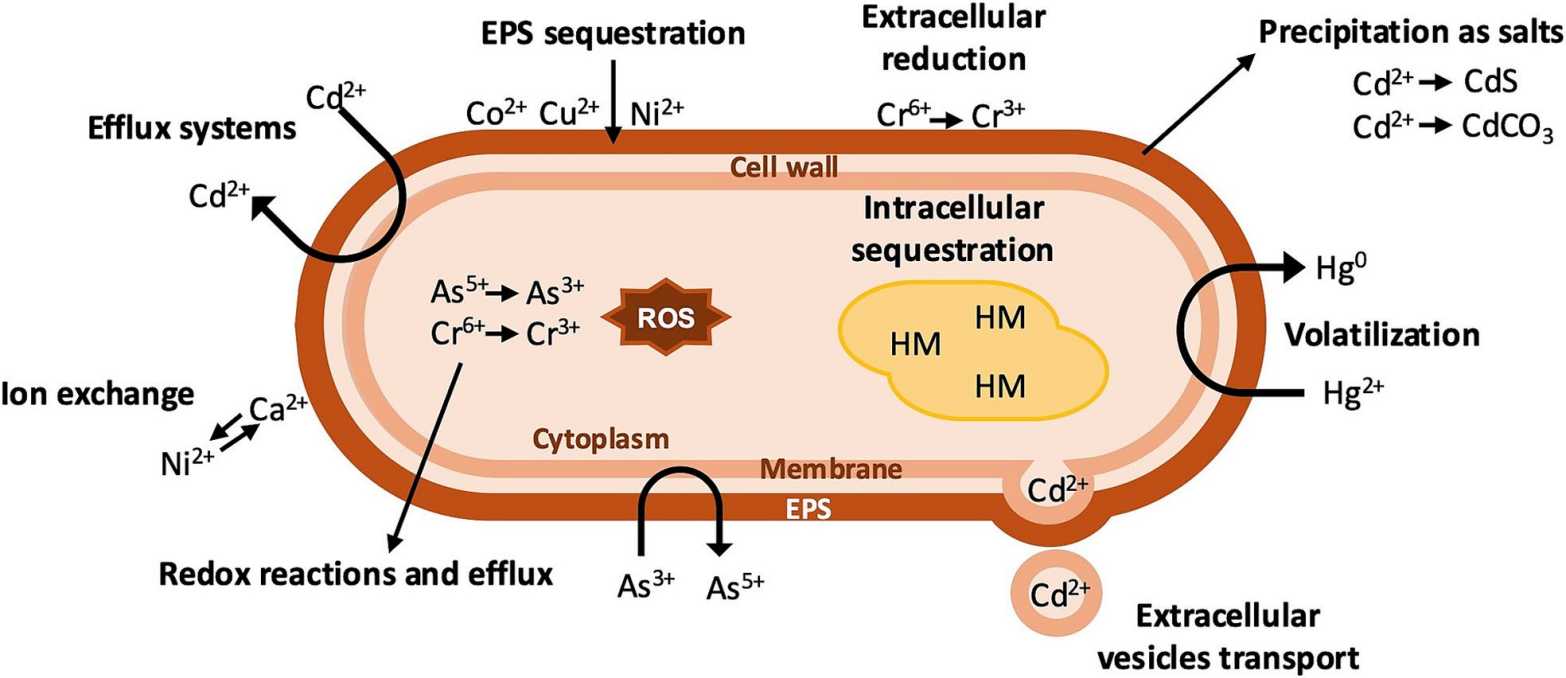
Mechanisms of HM resistance in bacteria. The reduction of HM to less toxic forms, production of metallothionein-like proteins, and intracellular sequestration occur in the cytoplasm. Salt precipitation, volatilization, ion exchange, metal efflux via efflux pumps, and extracellular sequestration occur on the cellular membrane and the EPS matrix.

In the environment, HM and metalloids primarily accumulate as positively charged cations (Priyadarshanee and Das, 2021), which microorganisms subsequently immobilize or transform into less harmful forms (Rana et al., 2021). Different bacterial species exhibit resistance to different HM concentrations (Table 2) through various mechanisms (Figure 1).

**Table 2:** Minimum inhibitory concentration (mM) and resistance levels to some of the most hazardous heavy metals and metalloids in different bacterial strains isolated from contaminated environmental samples.

Type of metal	Level of resistance	MIC mM	Bacterial strains	Sampling site	References
Cu(II)	Low	0.1**	*Escherichia coli*	Gut microbiota	Elguindi et al. (2009)
	Medium	4*	*Serratia plymuthica*	Soil contaminated with high arsenic level	Zanetti et al. (2022)
	High	70**	*Bacillus cereus*	Soil contaminated with contaminated wastes	Moeini et al. (2024)
Ni(II)	Low	1*	*Serratia plymuthica*	Soil contaminated with high arsenic level	Zanetti et al. (2022)
	Medium	2**	*Halobacterium salinarum*	Saltern soil	Baati et al. (2020)
	High	17**	*Pseudomonas aeruginosa*	Soil contaminated by nickel plating wastes	Devika and Pillai (2020)
Cd(II)	Low	0.5*	*Bacillus cereus* N5	Rhizosphere soil and roots of maize plant irrigated with industrial and municipal wastewater.	Abedinzadeh et al. (2019)
	Medium	3.1**	*Pseudomonas mendocina* KM594393	Soil samples derived from the heavy metalcontaminated paddy felds	Adhikary et al. (2019)
	High	18**	*Enterobacter mori* HZ21	Root nodules of *Robinia pseudoacacia*	Fan et al. (2018)
Cr(VI)	Low	1.5*	*Bacillus velezensis*	Chromium residue waste	Bao et al. (2022)
	Medium	6*	*Ensifer adhaerens*	Soil contaminated with high arsenic level	Li et al. (2022)
	High	9*	*Acinetobacter junii* B2w	Water samples	Sevak et al. (2023)
As(V)	Low	5*	*Arthrobacter scleromae* NM3E2	Roots of *Prosopis laevigata* and *Spharealcea angustifolia* grown in a heavy metal-contaminated	Román-Ponce et al. (2018)
	Medium	20**	*Pseudomonas mosselii* S6	Ice rhizosphere soil samples collected from an As contaminated paddy field	Xiao et al. (2020)
	High	100*	*Microbacterium arborescens* NE1E7	Roots of *Prosopis laevigata* and *Spharealcea angustifolia* grown in a heavy metal-contaminated	Román-Ponce et al. (2018)
	Very high	350*	*Bacillus tequilensis* ART2	Paddy fields	Pandey et al. (2020)
As(III)	Low	3	*Pseudomonas mendocina* KM594399	Soil samples derived from the heavy metal contaminated paddy felds	Adhikary et al. (2019)
	Medium	10*	*Micrococcus luteus* NE2E1	Roots of *Prosopis laevigata* and *Spharealcea angustifolia* grown in a heavy metal-contaminated	Román-Ponce et al. (2018)
	High	70*	*Alcaligenes* sp. Dhal-L	Arsenic contaminated soil	Cavalca et al. (2013)
Zn(II)	Low	0.5*	*Aeromonas media*	Heavy metal and petroleum hydrocarbon contaminated soil	Melzi et al. (2024)
	Medium	3*	*Enterobacter cloacae*	Rhizosphere soil and roots of maize plant irrigated with industrial and municipal wastewater	Abedinzadeh et al. (2019)
	High	15**	*Brevibactirium* sp.	Soil of an abandoned zinc mine	Taniguchi et al. (2000)
Pb(II)	Low	2**	*Lysinibacillus varians* KUBM17	Rhizospheric soil contaminated with industrial, sewage or agrochemical waste	Pal and Sengupta (2019)
	Medium	4**	*Burkholderia* sp. D54	Soil samples derived from the heavy metal-contaminated paddy fields	Guo et al. (2024)
	High	11**	*Serratia liquefaciens* H12	Rhizosphere soil of pakchoi in Cd-and Pb-contaminated farmland	Han et al. (2020)

*Mineral media.

**Rich media.

Typical mechanisms of HM resistance include extracellular and intracellular sequestration, detoxification via ion efflux or extracellular vesicle-mediated transport, and enzymatic detoxification (He et al., 2023).

Microbial extracellular HM sequestration primarily relies on adsorption, a metabolism-independent mechanism (Rana et al., 2021). This process involves both physical interactions, such as electrostatic or van der Waals forces, and chemical interactions that include ion exchange, complexation, diffusion, surface adsorption, and precipitation (Priyadarshanee and Das, 2021). For example, *Serratia plymuthica* strain As3-5a(5) isolated from As contaminated soil produces extracellular polymeric substances (EPS) and removes up to 91.5% of Cu(II) from a 200 mg L^−1^ solution, with a maximum adsorption capacity of 80.5 mg g^−1^ (Zanetti et al., 2022). EPS are mainly composed of polysaccharides and proteins, characterized by negatively charged functional groups including hydroxide (−OH), carboxylate (−COO), amino (−NH), and carbonyl (C = O), which play a crucial role in binding HM cations (Priyadarshanee and Das, 2021). Surface complexation takes place when metal ions form complexes with functional groups on EPS surface. An investigation on biosorption mechanisms identified the anionic carboxyl group as the primary binding site for cationic Cu(II) adsorption. Moreover, divalent cations like Ca^2+^ and Mg^2+^ interact with the EPS surface through ion exchange. This mechanism was observed in *Deinococcus radiodurans*, to remove up to 35% of Co(II) and 25% of Ni(II) in the presence of 25 mM Ca^2+^ (Syed et al., 2023). One of the major issues is EPS susceptibility to be lost during adsorption and separation processes. The inhibitory impact of HM on the bacterial biofilm might be due to water channel disruption across the biofilm for nutrient delivery. Also, chemical compounds generated after interaction with HM can directly disrupt the EPS layer and give an antibacterial effect (Syed et al., 2021). Several researchers have shown that negatively charged functional groups/ligands of EPS serve as a trap for HM ions. Enzymatic activities within EPS detoxify HM by transformation and successive precipitation in the polymeric mass (Pal and Paul, 2008; Haque et al., 2021). Hence, EPS yield, activity, and recovery remain crucial objectives to improve HM adsorption and facilitate EPS practical application (Li et al., 2022).

Another significant extracellular mechanism for metal binding is precipitation. Here, metal ions interact with functional groups found on the bacterial surface, leading to the formation of insoluble organic metal precipitates that adhere to microbial cells. Yan et al. (2024) studied two strains of *Burkholderia* sp. and *Bacillus* sp. to promote the conversion of absorbable Cd to CdCO_3_ via microbially induced calcium precipitation.

Intracellularly, metal ions can be sequestered by polyphosphate granules, glutathione, pyoverdine and cysteine-rich proteins, such as metallothionein, which exhibit high affinity for free metal ions (Demarco et al., 2023). Roy et al. (2024) found that *Pseudomonas aeruginosa* was able to accumulate Zn(II) ions inside the cell with high stability through the production of “Pseudomonas metallothionein A” (PmtA, Maltz-Matyschsyk et al., 2024).

Extracellular export through vesicles has gained recognition as a key mechanism in bacterial HM detoxification and homeostasis. Nano-sized membrane vesicles actively export toxic metal ions, reducing intracellular accumulation and increasing metal-induced stress. Recent studies have demonstrated that *Stenotrophomonas* sp. strain SH225 uses extracellular vesicles to sequester and expel Cd (Wang et al., 2023). Likewise, extracellular vesicles-mediated Cu secretion has been observed in other bacterial species such as *Synechocystis* sp. PCC6803, further supporting the idea that this process is a widespread adaptive strategy for metal efflux (Lima et al., 2022). By modulating metal transport and redistribution, bacterial extracellular vesicles not only contribute to microbial resilience, but also influence the environmental mobility and bioavailability of HM, with potential implications for bioremediation strategies.

Microbial enzymatic detoxification carried out by dioxygenases, peroxidases, and oxidoreductases, is essential in regulating the chemical biotransformation of HM into less toxic forms. This mechanism involves enzymes that catalyze redox reactions by altering the metal’s oxidation state (Haque et al., 2022). For instance, Demarco et al. (2023) reported that a *Bacillus* species resists Hg toxicity by mercuric reductase (MerA) that reduces Hg^2+^ to volatile Hg^0^ (Demarco et al., 2023; Priyadarshanee et al., 2022). Arsenic resistance operons ARS and AIO are found in bacteria and encode transport proteins, oxidoreductases, methyltransferases, efflux pumps, and transcriptional repressors. Arsenite (As(III)) oxidation to arsenate (As(V)) through arsenite oxidases (AioA, ArxA) and As(V) reduction to As(III) are widespread pathways to decrease As toxicity (Cavalca et al., 2013). Some bacteria utilize the As(III) S-adenosylmethionine methyltransferase, encoded by *arsM*, to transform As(III) to the less toxic methylarsenite, MAs(III) (He et al., 2023).

Some bacteria belonging to the genera *Rhodococcus* convert Cr(VI) to the less toxic Cr(III) intracellularly, through chromate reductase enzymes, relying on NADPH/NADH to donate an electron to Cr(VI) (Dubey et al., 2024). The presence of Cr(VI) leads aerobic bacteria to produce higher concentrations of reactive oxygen species (ROS) by competing with NADPH for electron binding. The increased production of ROS compels bacteria to release ROS-scavenging enzymes, such as catalase (CAT), glutathione S-transferase (GST) and superoxide dismutase (SOD) (Ramli et al., 2023). In *Shewanella oneidensis* MR-1, Cr(VI) reduction occurs extracellularly through the outer membrane cytochromes MtrC and OmcA, which facilitate electron transfer directly to Cr(VI) outside the cell, resulting in extracellular precipitation of Cr(III) (Shi et al., 2012; Beblawy et al., 2018).

## 3 Relation of biofilm-EPS genesis with QS

The highly coordinated multicellular behavior of microorganisms often leads to the formation of biofilms, which are aggregations of microbial cells adhered to a surface or to each other. Within biofilms, microorganisms coexist and mutually benefit from the development of diverse ecological niches over time. In biofilms, the microbial cells are lodged in a self-made matrix made of EPS, which contain large quantities of polysaccharides, proteins, and extracellular DNA. EPS facilitate bacterial adhesion to both biotic and abiotic surfaces, primarily driven by Van der Waals forces and hydrophobic interactions mediated by hydrogen bonding (Syed et al., 2023). The biofilm matrix provides protection against adverse environmental conditions, including fluctuations in nutrient availability, in salt concentrations, pH and temperature, and exposure to toxic chemicals (Syed et al., 2023).

Excessive HM concentrations can compromise the integrity of both the biofilm matrix and the embedded cells, indicating a threshold beyond which the protective capacity of these systems may be overwhelmed (Ding et al., 2019). For instance, studies on *Shewanella* species for water decontamination demonstrated that the effectiveness of biofilms in mitigating Cr(VI) toxicity is concentration dependent. While low levels (0.1 mM) of HM can be effectively managed by biofilm activity, higher concentrations can damage both the structure of the biofilm and of the bacterial cells (Ding et al., 2019). Similarly, in *Bacillus cereus*, which is beneficial for rice cultivation in Cd-contaminated soils, low concentrations (0.1 mM) biofilm structures remain intact, trapping HM, higher concentrations (0.2 mM) of Cd(II) cause oxidative stress, damaging cell membranes, and biofilm integrity (Jabeen et al., 2022).

**Figure 2:**
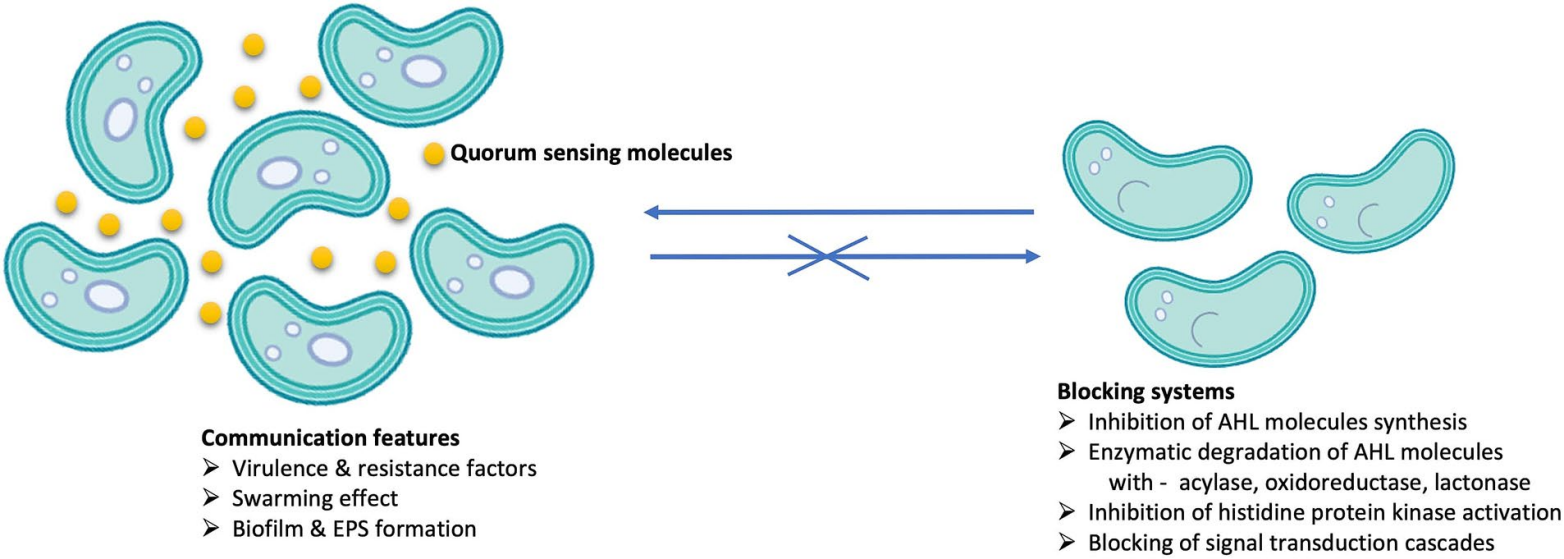
Processes that occur in the presence (left) and in the absence (right) of QS signal molecules (yellow circles) in microbial populations.

Within biofilms, the phenomenon of QS plays a crucial role. QS is defined as a decentralized process that, in complex communities, mediates microbial interactions by regulating gene expression in response to changes in the cell population density with the help of minute diffusible signal molecules called autoinducers (AI) (Shukla et al., 2014). Consequently, bacterial cell behavior varies depending on the cell density threshold. QS regulates several processes (Manzoor and Kaware, 2022) such as: attachment of cells to the surface, chemotaxis, production of EPS, symbiosis, biofilm formation, competence, conjugation, synthesis and secretion of biosurfactants and antibiotics, sporulation, bioluminescence and virulence, catabolic gene expression for the degradation of toxic compounds (Mangwani et al., 2014) and HM removal (Priyadarshanee and Das, 2021) (Figure 2).

Recent studies have revealed that CRISPR-Cas systems can both suppress and promote biofilm formation by modulating QS pathways. Guo et al. (2025) successfully applied high-efficiency CRISPR-Cas9 genome editing in *Bacillus velezensis* FZB42, a plant growth-promoting bacterium and biocontrol agent. By targeting the *slrR* gene, a key regulator of biofilm development, they increased biofilm formation. These findings highlight the promising role of CRISPR-Cas technology for sustainable agriculture. By expanding gene editing to HM resistant strains, it will be possible to improve the efficiency of environmental biotechnology.

**Figure 3:**
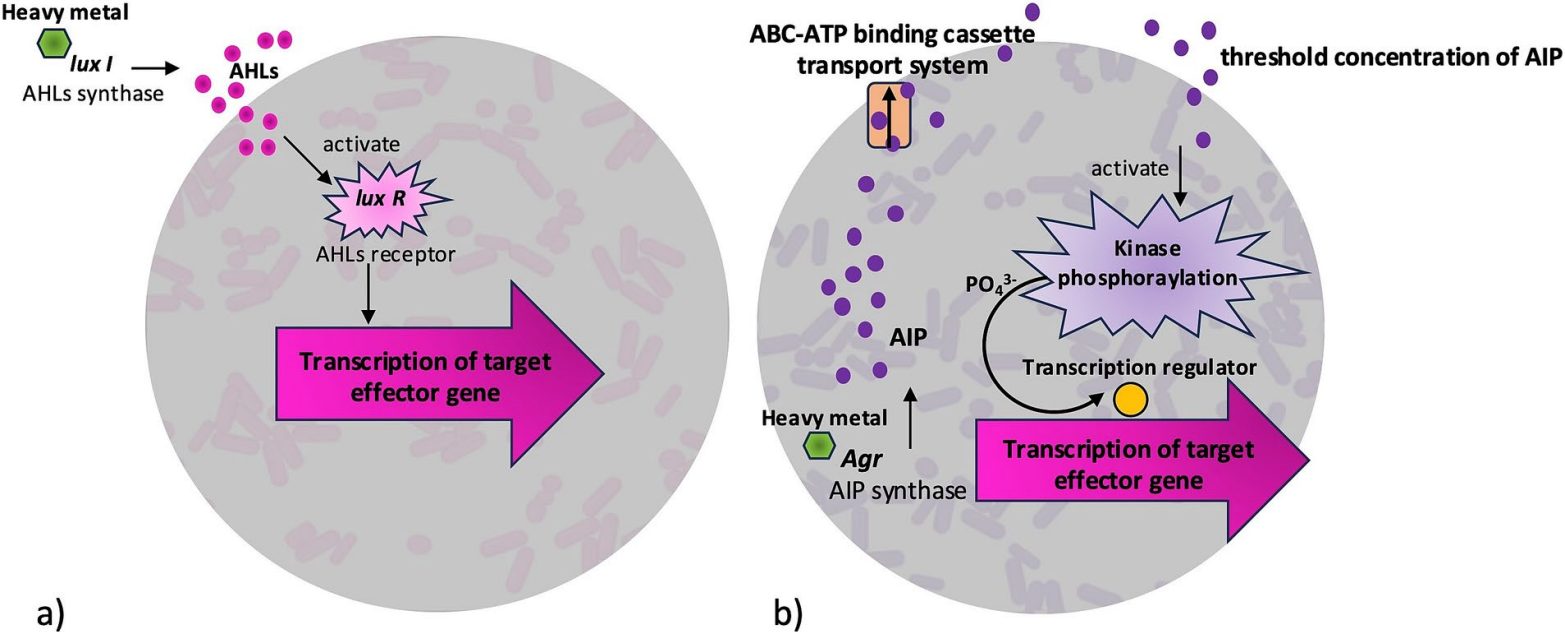
Quorum sensing-dependent gene regulation that can be triggered by heavy metals. In Gram-negative bacteria **(a)**, LuxI catalyzes the synthesis of N-acyl homoserine lactones (AHLs) synthase to produce AHLs signals, which bind to the LuxR receptor. The AHL-LuxR complex then activates transcription of target effector genes. In Gram-positive bacteria **(b)**, Accessory gene regulator (Agr) stimulates the synthesis of auto-inducing peptides (AIP). When AIP reach a threshold concentration, they activate a kinase, leading to phosphorylation of a transcription regulator that initiates transcription of target genes.

Biofilm bacteria react to changes in nutrient gradient, oxygen availability, and harsh environmental conditions by changing their gene expression pattern and physiological function with the help of signaling molecules inside the matrix (Mitra et al., 2014). In bacterial biofilm, high cellular densities trigger the production of AI both within and among bacterial species. When a minimal threshold concentration is reached, AI regulate gene transcription leading to formation and development of biofilms best adapted to environmental conditions (Donato et al., 2016; Mohapatra et al., 2019). Three types of AI are found widely in bacteria: *N*-acyl homoserine lactones (AHLs), autoinducing peptides (AIP), and autoinducer-2 (AI2). Gram-negative bacteria employ the most studied signaling system based on AHLs, gram-positive bacteria use AIP (Mangwani et al., 2012) (Figure 3). In gram-negatives, AHLs synthase interacts with reporter LuxI protein, leading to the diffusion of AHLs across the cellular membrane. AHLs increase with cell density, causing the signal to interact with receptor protein LuxR, thus controlling expression of target effector genes for biofilm formation (Figure 3a). In gram-positives AIP-based signaling, the accessory gene regulator Agr activates AIP synthase to produce small linear or cyclic peptides. During secretion by ABC-ATP binding cassette transport system, peptides are assembled as large pro-peptides and detected by a membrane-bound protein sensor (Figure 3b). When concentration threshold is achieved, kinase-mediated phosphorylation activates transcription of effector genes for biofilm formations (Donato et al., 2016; Mukherjee and Bassler, 2019). The less studied LuxS/AI2 QS system is involved in interspecies communication both in gram-positive and gram-negative bacteria (Manzoor and Kaware, 2022).

## 4 QS regulation in the presence of HM

HM accumulated in the environment are positively charged and can absorb onto the negatively charged sites of biofilm by electrostatic attraction. The complex mixtures of glycocalyx matrices created by bacterial species result in a crosstalk between QS molecules. It can be hypothesized that the biofilm environment may provide a protective milieu for bacterial cells to thrive under multi-metal stress that may add increased sequestration and immobilization of metal ions (Syed et al., 2021). Metal toxicity interrupts the structural and functional properties of enzymes by the binding of thiol and protein groups, or by replacing cofactors in the prosthetic group (Manzoor and Kaware, 2022). *Acinetobacter junii* strain BB1A, a novel metal-tolerant bacterium, form biofilms in presence of Ni^2+^, AsO_2_^−^, Cd^2+,^ and Hg^2+^ on surfaces such as glass and polystyrene. The association of metal tolerance with the production of biofilm has been confirmed by the generation of a metal-sensitive and adhesion-deficient mutant by insertion of Tn5-mob mutation (from plasmid pSUP5011) in the *Acinetobacter junii* genome. The necessity of a critical cell density for biofilm formation and the presence of AHLs in the cell-free supernatant indicated the capacity of QS (Sarkar and Chakraborty, 2008). It was demonstrated that Ni and Cd can inhibit biofilm formation in *Burkholderia multivorans* by disrupting homoserine lactone QS, without affecting cell viability (Vega et al., 2014). Another study showed that, in the presence of sub-lethal concentrations of Cd salts, QS was inhibited in *Chromobacterium violaceum*. Notable inhibition was seen against pigmentation, motility, chitinase production, and biofilm formation with repression of QS-signaling genes at the level of transcription (Thornhill et al., 2017). Gene expressions of *afeI* and *afeR* (predicted like LuxI-LuxR proteins) in *Acidithiobacillus ferrooxidans* has been studied, given its high tolerance to HM like Cu^2+^. The observation of a significant reduction in the tolerance of Cu^2+^ ions in the presence of furanone compounds (molecules known to interrupt QS) firmly suggested the vital role of QS in HM resistance. Moreover, it has been suggested that AHL mediated QS regulate the ability of these microorganisms to utilize energy (Montgomery et al., 2013). These observations demonstrate that HM can also act as triggers of QS signaling expression, as shown in Figure 3.

In the presence of Cd(II) and Pb(II) in wastewater, exogenous AHL mediated QS significantly enhanced their removal, with the average removal efficiency increasing by 36.3 and 37.2%, respectively. AHL also promoted the secretion of EPS, carbohydrate transport, and stimulated microbial metabolism and antioxidative response, which improved removal of pollutants in activated sludge (Zeng et al., 2024). HM, as a widespread environmental stressor, can significantly impair bacterial function, particularly affecting anaerobic ammonium oxidation (anammox) consortia. Anammox consortia mainly depend on three communication signals for the operation of QS system: intraspecific AHL signals, intra-and interspecific diffusive signal factor (DSF) signals – encoded gene *RpfF*, and interspecific AI-2 signals (Guo et al., 2024; Zhang et al., 2023; Qu et al., 2025). These regulate the expression of downstream genes to strengthen anammox performance. Metabolomics analyses revealed that AHL impacted bacterial activity by regulating electron shuttles, boosting bacterial growth through modulation of glycerophospholipid metabolism, and promoting floc aggregation. These metabolic changes induced by exogenous AHL led to significant improvements in the specific anammox activity, nitrogen removal rate, and growth rate of anammox consortia during the anammox process. Previous research demonstrated that QS regulates EPS production in the anammox system. 2 μM AHLs exogenously increased the levels of extracellular proteins in the EPS of anammox consortia by 21–34% and polysaccharides by 6–17%. The increased EPS, regulated by QS, enhanced the biosorption of HM (Pagliaccia et al., 2022). A single-metal biosorption study revealed that the adsorption capabilities of EPS extracted from anammox consortia were 84.9, 52.8, 21.7, and 7.4 mg·gTSEPS^−1^ for Pb(II), Cu(II), Ni(II), and Zn(II), respectively. In addition, Tang et al. (2024), demonstrated that the role of AHL in extracellular partitioning and complexation is not significant to resist Cu(II) stress, whereas its primary function is to enhance intracellular resistance. Particularly, AHL regulates the production of superoxide dismutase (SOD) and catalase (CAT), strengthening intracellular antioxidant capacity. AHL addition significantly reduced fluorescence intensity by 29% compared to the control without AHL, demonstrating its role in scavenging intracellular ROS under Cu(II) stress. Furthermore, AHL modulates cysteine biosynthesis, enabling Cu(II) sequestration via its thiol ligand within the cells, and regulates efflux pump proteins to facilitate Cu(II) export.

**Figure 4:**
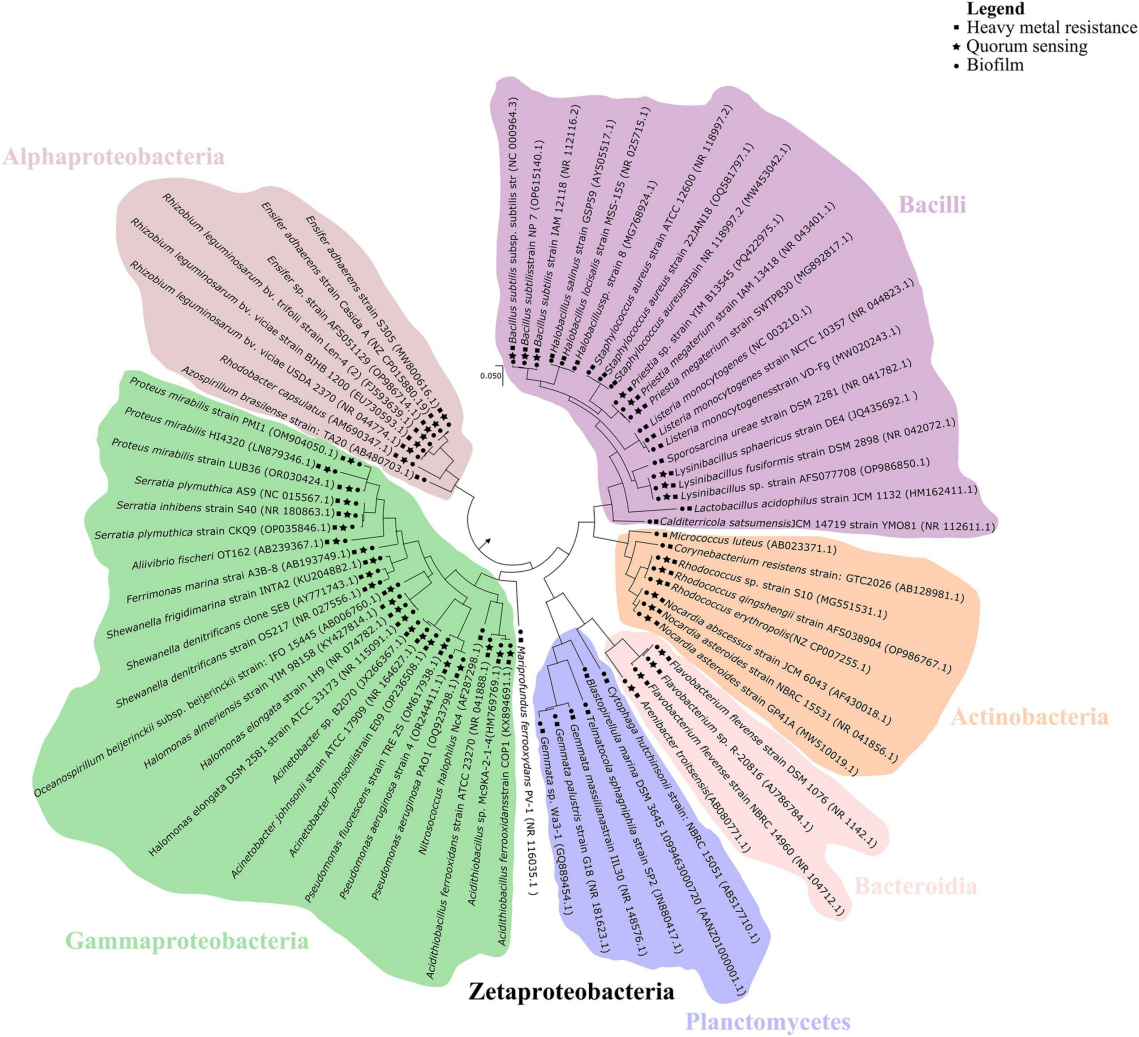
Phylogenetic distribution of quorum sensing (stars), biofilm formation (circles), and heavy metals resistance (squares) genes across bacterial taxa. The phylogenetic tree illustrates the evolutionary relationships among representative bacterial species, with major taxonomic groups color-coded for clarity. Phylogenetic affiliations were determined using MEGA11 (Tamura et al., 2021) based on bacterial genome sequences retrieved from the NCBI database. Evolutionary history was inferred using the Maximum Likelihood method with the Tamura–Nei model (Tamura and Nei, 1993). The tree is drawn to scale, with branch lengths representing the number of substitutions per site.

The phylogenetic distribution of QS, biofilm formation, and HM resistance genes suggests a potential evolutionary link between these adaptive mechanisms (Figure 4). QS and biofilm-associated genes often co-occur with HM resistance determinants, particularly within the bacterial classes Alphaproteobacteria, Gammaproteobacteria, Zetaproteobacteria and Bacilli. The widespread co-occurrence of these genes may reflect an adaptive mechanism. QS and biofilm formation enhance bacterial resistance to HM, thus improving microbial survival and ecological fitness. Interestingly, QS genes have not been detected within the phylum *Planctomycetota*, in accordance with previous studies (Rehman et al., 2019). However, since these microorganisms commonly exist in a biofilm lifestyle, the hypothesis is that genes with function analogous to the well-characterized QS genes may be present but have yet to be described.

## 5 Biofilm dynamics in HM-affected environments

In nature, microorganisms tend to live in biofilms as a strategy that increases their survival rate. In fact, biofilm lifestyle provides a higher resistance to various environmental stresses if compared to planktonic lifestyle (Harrison et al., 2007; Parrilli et al., 2022) and leads to closer interaction between cells.

HM exposure occurs in biofilms inhabiting several ecosystems both due to natural and anthropogenic sources (Table 3). Despite the high toxicity of HM, prior studies have shown that the biodiversity of microbial communities found in contaminated environments is more influenced by physicochemical parameters like pH, temperature, and nutrient availability than by HM (Drewniak et al., 2016). In a study performed in Deûle river (France), Jacquiod et al. (2018) hypothesized that the presence of HM selects HM-resistant microorganisms while preventing the growth of HM-sensitive opportunists.

**Table 3:** Microbial communities of natural biofilms living in contaminated environments.

Type of ecosystem	Location	Contamination	Biomarkers	References
Acid mine drainage	Richmond mine, Iron Mountain (California)	HM	Metagenome, metaproteome	Tyson et al. (2004)
Acid mine drainage	Mynydd Parys (UK)	HM	Prokaryotic 16S rRNA genes, eukaryotic 18S rRNA genes, esterases-encoding genes	Kelly et al. (2023)
Acid mine drainage	Carnoulès (France)	As	Metagenome, metaproteome	Bertin et al. (2011)
Acid mine drainage	Drei Kronen und Ehrt (Germany)	HM	RFLP	Ziegler et al. (2009)
Acid mine drainage	Guangdong Province (China)	HM	Metagenome, metatranscriptome	Chen et al. (2015)
Acid mine drainage	Sherlovaya Gora polymetallic mine, Chita oblast (Russia)	HM	Metagenome	Kadnikov et al. (2016)
Acid mine drainage	Los Rueldos (Spain)	HM	Prokaryotic 16S rRNA genes, eukaryotic 18S rRNA genes, esterases-encoding genes	Mesa et al. (2017), Vidal et al. (2022)
Mine tailing	Dexing copper mine (China)	HM	Metagenome, metaproteome	Zhang et al. (2016)
Acid mine drainage-affected streams	Zloty Stok and Kowary mines, Lower Silesia (Poland)	HM	Metagenome	Drewniak et al. (2016)
Acid mine drainage-affected river	Rio Tinto (Spain)	HM	EPS, metatranscriptome	Aguilera et al. (2008), Moreno-Paz et al. (2010)
River	Sensée Canal and DeÛle river, Férin (France)	HM	Metagenome, metaproteome	Gillan et al. (2015), Jacquiod et al. (2018)
River	Chaizhibang river, Changzhou, (China)	Urban pollutants	DNA GeoChip hybridization	Sun et al. (2022)
River	Qiantang River (China)	Urban pollutants	Bacterial 16S rRNA genes	Geng et al. (2022)
Sea	Hongtang Bay, Sanya (China)	Cu, Zn	Bacterial 16S rRNA genes, metagenome	Zhang et al. (2019)
Biological aqua crusts	Meizhou, Guangdong Province (China)	Pb, Zn	Metagenome	Wang et al. (2023)

For example, in Acid Mine Drainage (AMD)-affected environments, the low pH (usually ~ 2) is the major driver that, together with HM, shape the microbial communities leading to a lower diversity compared to neutral-pH sites (Méndez-García et al., 2015). AMD are typically oligotrophic environments, where biofilms are established around primary producers and nitrogen fixers that provide a suitable environment for organic carbon degraders, leading to the development of a more complex community driven by few microbial species (Tyson et al., 2004; Moreno-Paz et al., 2010). Notably, the activity of heterotrophic microorganisms enables the coexistence of autotrophic microorganisms by eliminating dissolved organic carbon, which acts as an inhibitor for autotrophic microorganisms (Méndez-García et al., 2015). In these environments, the bioenergetic electron chain is usually dependent on the presence of O_2_ and Fe(III) oxides as electron acceptors and of Fe(II) and sulfides as electron donors, although the presence of metals and metalloids such as As promotes the presence of metal-respiring microorganisms (Tyson et al., 2004; Bertin et al., 2011; Chen et al., 2015). A metaproteomic study performed on sediments of the AMD-affected river Reigous (Carnoules mine, France), revealed that stress derived from the exposure of microorganisms to HM triggers the expression of signal molecules that leads to the formation of microbial cell structures such as pili involved in twitching motility and adhesion, crucial for biofilm formation (Bertin et al., 2011). This phenomenon has been reported also in the planktonic microorganisms inhabiting the water of different mine sites in China (Chen et al., 2015), although in the extremely acidic river Rio Tinto (Spain) an up-regulation of genes involved in biofilm formation (*mqsR, cheAY, fliA, motAB*) has been detected in biofilm cells in comparison with planktonic cells (Moreno-Paz et al., 2010). A metagenome-scale analysis in the biofilms living in Dexing Copper Mine (China) revealed the presence of genes involved in different types of signaling pathways, such as signal transduction, secretion, and vesicular transport (Zhang et al., 2016).

A study of Biogenic Aqua Crusts (BAC) in an abandoned Pb/Zn tailing pond in China revealed that within complex biofilms made of both heterotrophic and autotrophic members, autotrophic/diazotrophic microorganisms such as Leptolyngbyaceae spp. were responsible for the up-regulation of genes encoding biofilm formation proteins (i.e., OmpR, CRP and LuxS) (Wang et al., 2023).

Overall, the above-mentioned studies suggest that in the environment, microbial HM exposure promotes biofilm formation as a strategy to cope with toxicity. However, a significant knowledge gap exists concerning which metabolic pathways involved in HM resistance and transformation are triggered by QS and concerning the role of each microbial species that constitutes the biofilm in these mechanisms. A higher effort in metagenomic and metatranscriptomic studies performed in natural contaminated sites should cover this gap, clarifying the pathways present and expressed in the biofilms exposed to HM in different conditions (i.e., temperature, light exposure, pH, hydration, etc.).

## 6 QS-based mechanisms in HM bioremediation

Both planktonic-and biofilm-based cell systems can be exploited for environmental biotechnology application, though biofilms have been more extensively studied, since biofilm-specific physical and biochemical mechanisms are involved in metal bioaccumulation, binding or transformation. Moreover, QS communication within bacterial communities can be exploited in bioremediation and metal biosensing technologies, since experimental data support its role in the expression of HM resistance mechanisms (Teitzel and Parsek, 2003; Hsu et al., 2016).

Biotransformation mechanisms play a crucial role in HM resistance and detoxification in microbial communities. For example, the induction of Cu resistance and removal was studied in Anammox consortia, to assure nitrogen removal in metal-contaminated wastewater. Tang et al. (2024) found that AHL incremented Cu resistance in Anammox consortia by upregulating intracellular metal resistance mechanisms and antioxidant ROS scavenging systems, rather than by metal extracellular partitioning and complexation. Similarly, Qu et al. (2025) demonstrated that AHL-mediated QS contributed to metal resistance by modulating electron transfer pathways and facilitating the transformation of As(III) into less toxic redox states As(V) and monomethylarsonic acid.

Bacterial biofilms can effectively remove metals through bioaccumulation due to their high biomass density and can also reduce the toxicity of certain metals through enzymatic activity. The polyionic characteristics of biofilm EPS enable the binding of metal ions, leading to the formation of organometallic complexes through electrostatic attraction (Mohapatra et al., 2019). Specifically, microorganisms involved in bioaccumulation use proteins to uptake and sequester metal ions in the intracellular space to utilize in cellular processes, including QS molecules that affect the bioavailability and transportation of metals (Sharma et al., 2022). Adsorption technology represents an advantage because it is a passive process adaptable to higher metal concentrations and to different environmental conditions, without the need of nutrient supplementation for growing cells. The efficacy of biosorption is primarily dependent on the characteristics of the biosorbent and the specific HM present in the environment. It was recently demonstrated that Ni and Cu adsorptions were governed by different kinetic models explicated by the different outer cellular structures in two *Serratia plymuthica* strains (Zanetti et al., 2022). Protonation of HM is the predominant characteristic that can vary its susceptibility to binding (Ciani et al., 2024). Living bacterial biomass rely their sorption ability on biofilm EPS structures that enhance cell interactions, protect biofilm structures, while determining HM removal according to a composite biochemical framework (i.e., polysaccharides, proteins and nucleic acids). Emerging evidence suggests that sub-inhibitory concentrations of HM, including Cd, Pb, and Ni, can stimulate biofilm formation in various microorganisms. For instance, Giovanella et al. (2016) reported enhanced biofilm production by *Pseudomonas* sp. isolates in the presence of Hg. In different strains, short-chain QS sensing molecules (acyl-homoserine lactone) were found to positively regulate Cd resistance and higher removal efficiency via the formation of EPS-mediated biofilm in *Aeromonas diversa* strain VITKKAJ1 (Itusha and Mohanasrinivasan, 2018). According to these findings (Sun et al., 2024) demonstrated that in *Ensifer adhaerens* NER9 strain the QS SinI/R system located on the linear chromosome could affect the interactions between functional groups on cell surfaces and Cd, EPS production, and HM resistance contributing to bacterial Cd biosorption in solution via cell wall and EPS adsorption.

The study (Nguyen et al., 2014) of the structure of fusion protein CgsA suggested that these extracellular self-assembled amyloid nanofibers confer strong adhesion ability to biofilms, leading to the development of artificial peptides that constitute nanofibers more effective in binding HM thus aiding in bioremediation (Chen et al., 2015).

Microbial bioflocculants are extracellular polymers secreted by microorganisms that contribute to flocculating activities as they provide binding sites to pollutants. Due to the negative charge of microbial bioflocculants, polymer bridging has been suggested as the main flocculation mechanism (Selepe and Maliehe, 2024). N-dodecanoyl-l-homoserine lactone (C12-HSL), which serve as autoinducers for QS regulation in Gram-negative bacteria, have demonstrated positive effects on the production of aromatic proteins in EPS during bioflocculation. *Chlorella*-bacteria system secreted tryptophan and extracellular aromatic proteins as the main components in EPS of bioflocs during fed-batch cultivation. The result indicated that exogenous C12-HSL induced *Chlorella* to secrete aromatic proteins for microalgal−bacterial bioflocculation, which is beneficial to microalgae-based wastewater treatment (Wang et al., 2022).

Over the past decade, there have been many discussions about leaching biofilms, where the attachment of cells to solid surfaces is a crucial step in initiating bioleaching, as it leads to the formation of the biofilm, which is vital to the mineralization process (Luo et al., 2024). Following the process, the impact of overexpression of the endogenous QS machinery (qs-I operon/afeI gene) on the covellite bioleaching capabilities of acidophilic chemolithoautotrophic bacterium, *Acidithiobacillus ferrooxidans* was explored. The strains exhibited increased transcriptional gene expression of afeI and enhancing cell adhesion to covellite, along with elevated production of extracellular polymeric substances and biofilm formation. It was observed that the strains show the potential of genetic modulation of QS to enhance the bioleaching efficiency of covellite, and other copper sulfide minerals (Jung et al., 2023). *Leptospirillum* spp. are effective mineral colonizers. *Leptospirillum ferriphilum* and *Leptospirillum ferrooxidans* demonstrated that it can produce (Z)-11-methyl-2-dodecenoic acid when grown with pyrite. The biomolecule is a functional diffusible signal factor involved within interspecies QS signaling mechanism. In addition, both pure diffusible signal factors and extracts from the supernatants of pyrite-grown inhibited biological iron oxidation. Furthermore, pyrite-grown *Leptospirillum* strains were less susceptible to self-inhibition compared to iron-grown cells (Bellenberg et al., 2021).

## 7 QS implementation in environmental biotechnologies

The above-mentioned scientific evidence has been used to develop QS-based strategies for enhancing HM resistance and transformation in bioremediation systems.

### 7.1 Biofilm bioreactors

Diverse biofilm reactors are reported to bioremediate HM. In anaerobic environment, biofilm reactors use sulfate reducing bacteria to scavenge metals and precipitate metal sulfides in contaminated water at the biofilm interface. It was evident that mixed species biofilm consisting also of sulfate-reducing bacteria, catalyzed the precipitation of metal sulfides of Cu, Zn, Ni, Fe, and As (Jong and Parry, 2003). This treatment resulted in removal of 98% of Zn, Cu, Ni, and 82 and 78% of Fe and As, respectively. But aerobic microbes grow on a rotating cylinder (Rotating Biological Contactors) and are used for treatment of water contaminated with volatile organic compounds, HM, dyes and polycyclic aromatic hydrocarbons (PAHs) (Jasu and Ray, 2021). Biofilm-based systems offer numerous advantages, such as enhanced pollutant removal efficiency, reduced sludge production, shorter hydraulic retention times, the presence of EPS, high levels of active biomass, and a diverse microbial community, while biofilm reactors consist of five main compartments and additional specific components tailored to each reactor type: a containment structure, influent wastewater with specified contaminant levels, biofilm substratum for microbial attachment and growth, a wastewater collection system, and a mixing or aeration system for carrier distribution and agitation. The types of biofilm bioreactor are: (1) Membrane biofilm reactor (2) Moving bed film reactor (3) Fluidized bed film reactor (4) Microbial fuel cells (5) Trickling filter (6) Granular sludge (Sahreen et al., 2022; Saini et al., 2023). However, simulation and modeling studies ought to be conducted for better design and optimization of removal processes. In a biofilm system composed of diverse strains, successive sorption–desorption cycles were appropriate to remove Cu, Zn, and Cd (Costley and Wallis, 2001). Mixed biofilm was able to retain Hg from contaminated wastewaters when used in packed-bed bioreactors, the ability was higher when compared to monoculture biofilm due to higher microbial diversity which was able to cope with rapidly changing mercury concentrations (Canstein et al., 2002). The overall biosorptive capacity of biofilms can be limited by the presence of bacterial species with lower resistance to HM (Morel et al., 2009).

Azizi et al. (2016) demonstrated the removal of composite toxic metal ions (Cu, Ni, Cd, and Zn) from effluent wastewater using a packed bed biofilm reactor (PBBR) biological system. A PBBR system is based on attached growth treatment technology, producing biofilms on supporting media, and showed efficient HM reductions of 90.32, 90.28, 87.9, and 82.14%, respectively, at different loading concentrations of composite HM of 8 mg/L, 20 mg/L, 28 mg/L, and 40 mg/L at an optimum HRT of 2 h. Furthermore, Grujić et al. (2017) demonstrated superior Hg removal of mixed-culture biofilms was achieved in these reactors compared to monocultures, highlighting the importance of microbial diversity. HM removal using biosorption/adsorption capacity of aerobic granules has been explored as a convenient method (Sengar et al., 2018). In comparison with bioflocs, granules possess much better settling ability and dense microbial structure. Further aerobic granules have shown excellent biosorption of many HM such as Ni, Cu, Zn, and Cr (Li et al., 2017; Wang et al., 2010; Wei et al., 2016; Yao et al., 2009).

Rotating biological contactors demonstrate the ability of biofilms to withstand repeated metal sorption–desorption cycles, indicating their potential for long-term operation. Priyadarshanee and Das (2021) reported the bioremediation efficiency of the biofilm-forming, metal tolerant *Pseudomonas chengduensis* PPSS-4 strain under various environmental conditions. PPSS-4 showed excellent biofilm formation ability at a higher concentration of 100 mg/L for Cr, Cd, and Pb, and showed maximum removal of 91.14, 74.5, and 92.91% of the respective metals in a batch culture, as compared to planktonic cells, which showed 85.35, 65.71, and 84.71% removal, respectively. The maximum removal of Cr, Cd, and Pb was achieved by a bacterial biomass at optimized environmental conditions of pH level (6.0), temperature (37°C), and salinity 4% for Cr and Pb, and 6% for cadmium, within 4 h. Further instrumental analysis with Field Emission Scanning Electron Microscopy coupled with Energy Dispersive X-ray Spectroscopy (FESEM-EDS) proved the sequestration of metal ions on the bacterial membrane surface and in the biofilm-EPS complex. Moreover, FTIR analysis demonstrated the interaction mechanism of chemical functional groups, like hydroxyl (−OH) and phosphate (P– O) and ammonium (−NH), with HM.

### 7.2 Biofilm integrated nanofiber display

Biofilms as self-assembled synthetic functional material, researchers have been able to design and utilize them for specific purposes. It is a nanobiotechnological platform that involves molecular programming of bacteria’s extracellular matrix through the addition of peptide domains, creating tailored structures for customized outputs. The proteinaceous component of the biofilm formed by bacteria are networks of amyloid nanofibers and retain the functions of peptide domain. By modifying the biofilm matrix using BIND, amyloid nanofibers play an altered role in biofilm stability, enhancing bacterial adhesion and aggression while aiming to confer artificial functions such as covalent immobilization of proteins, substrate specificity for adhesion, and nanoparticle bio-templating to create a more designable biomaterial for effective bioremediation. This interactive approach combines material science and synthetic biology to develop programmable living material capable of thriving in extremely metal-affected environments and aiding in subsequent remediation processes (Jasu and Ray, 2021).

### 7.3 Electroactive biofilms

In bioelectrochemical systems (BES), microbial extracellular electron transfer (MEET) is facilitated by the presence of solid-state electron acceptors/donors. MEET is carried out by electroactive microorganisms as a respiratory process to be coupled with other metabolic activities and can be utilized for eliminating organic and inorganic substances. MEET can occur both in exo-electrogenic microorganisms, that donate electrons to extracellular acceptors, and in electrotrophs, that accept electrons from extracellular matrices. Electroactive microorganisms play crucial roles in various bioremediation processes, including mineral recycling, energy production, biosensing, and pollutant removal (Logan et al., 2019; Chiranjeevi and Patil, 2020). Moreover, electroactive microorganisms are involved in the formation of electroactive biofilms (EABs) in wastewaters for bioremediation, in BES such as microbial fuel cells and microbial electrolysis cells. The development of EABs, influenced by factors such as bacterial species, temperature, pH, substrate pattern, and electrode composition, varies significantly. Mixed culture EABs, in contrast to pure culture EABs, not only produces more energy but also exhibits greater potential for MEET (Chattopadhyay et al., 2022).

## 8 Plant-microbiome communication in HM removal

Biofilm-forming microbes, including diverse bacteria, yeast, fungi, and symbionts, can both suppress plant pathogens and promote plant growth by regulating nutrient availability, depending on their location of colonization (Rayanoothala et al., 2020). In the densely populated rhizosphere biofilms, bacteria adapt their genetic expression by employing diffusible signal molecules to sense the density of their own surrounding bacterial populations. The ability of bacteria to adapt to fluctuating environments and successfully colonize eukaryotic hosts heavily relies on QS (Mukherjee and Bassler, 2019).

Rhizosphere ecosystems are pivoted by QS-mediated microbial/plant communication that shape microbial interactions within the rhizosphere (Tariq and Ahmed, 2023; Sharma et al., 2022). Bacterial AHLs are detected by plants and influence tissue-specific genes and defensive mechanisms (Daniels et al., 2004). AHLs mimic chemicals (e.g., furanone signals) generated by higher plants can govern QS-regulated bacterial population dynamics (Pérez-Montaño et al., 2013).

Researchers first demonstrated plant hosts ability to perceive QS signals by analysing *Medicago truncatula*’s response to AHL-QS signals (Mathesius et al., 2003) subsequently investigated how AHL application to tomato roots induced pathogen resistance (Hartmann et al., 2024). Symbiotic relationships, such as those between plants and microbes, offer numerous benefits, including enhanced tolerance to HM (Tripathi et al., 2021). Association between plant roots and bacterial biofilms has been investigated to be exploited as microbial-assisted phytoremediation, or on the other hand as means of food safety to reduce metal accumulation in edible plants (Majhi et al., 2023).

Plant growth promoting bacteria reduce metal stress for plants or increment their biomass, mainly by the reduction of stress ethylene via ACC deaminase, metal precipitation or by excluding metal to be uptake by EPS adsorption (Meryem et al., 2022; Dutta et al., 2022). Wang et al. (2025) evidenced that in rhizosphere soil of *Sedum alfredii* bacterial genes for most of signal molecule-synthesizing enzymes and EPS production, such as *trpE, trpG, bjaI, rpfF, ACSL,* and *yidC* were overexpressed in the presence of Cd and Zn. The plant promoted HM transfer from bulk to rhizospheric soil and accumulated HM in roots and leaves, demonstrating that HM increased the transcription of genes for both signal molecule and metal adsorbing surface layer production.

## 9 Conclusion

Our understanding of QS signalling microorganisms in contaminated environments remains limited, largely due to the complexity of QS systems, in relation to HM bioavailability and site-specific conditions. Further research is necessary to optimize the delivery of stable QS modulators and to assess potential ecological risks also in relation to genetically engineered microorganisms. Exploring the role of QS in plant-microbiome communication could inform strategies for improving root colonization by HM-resistant bacteria, enhancing plant fitness and optimizing HM uptake for more effective phytoremediation.

Advanced analytical techniques, such as omics-based approaches, single-cell analysis, synthetic biology and gene editing, could provide deeper insights into the role of QS in HM resistance and transformation both at cellular and environmental levels. Scaling QS-based bioremediation to real-word scenarios is challenging due to high production costs of QS modulators, and the need for reliable monitoring tools. Artificial intelligence (AI) and machine learning (ML) could be considered valuable tools for modelling QS networks, predicting microbial behavior. By analysing complex omics datasets, simulating environmental conditions, and optimizing parameters through nature-inspired algorithms, AI and ML can enable more accurate predictions of microbial interactions and interspecies communication.

Overcoming these knowledge gaps and practical limitations is crucial to unlocking the full potential of QS-based bioremediation as a sustainable environmental solution.
